# Online Videogames Use and Anxiety in Children during the COVID-19 Pandemic

**DOI:** 10.3390/children8030205

**Published:** 2021-03-08

**Authors:** Concetta De Pasquale, Matteo Chiappedi, Federica Sciacca, Valentina Martinelli, Zira Hichy

**Affiliations:** 1 Department of Education Science, University of Catania, 90124 Catania, Italy; depasqua@unict.it (C.D.P.); federica.sciacca@hotmail.com (F.S.); z.hichy@unict.it (Z.H.); 2 Developmental Psychopathology Research Unit, IRCCS Mondino Foundation, 27100 Pavia, Italy; 3 Department of Brain and Behavioral Sciences, University of Pavia, 27100 Pavia, Italy; valentina.martinelli@unipv.it

**Keywords:** videogames, internet gaming disorder, anxiety

## Abstract

Videogames use has constantly increased among children and adolescents, with uncertain consequences on their health. This study aimed to assess the prevalence of videogames use and addiction in a sample of Italian children during the COVID-19 pandemic and their association with anxiety symptoms. One hundred and sixty-two children (M/F:78/84; age range: 8–10 years; average age 9.4 ± 0.7 years) completed the Videogame Addiction Scale for Children (VASC), the Test of Anxiety and Depression (TAD) and the Children’s Anxiety Meter—State (CAM-S). Demographic variables and data on the access to electronic tools and games preferences were also collected. Overall, 96.3% of the study participants stated to have access to one or more device. They reported a low risk of videogame addiction (VASC score (mean ± SD): 46.7 ± 15.4), a moderate level of trait anxiety (TAD score (mean ± SD): 135 ± 16.8) and a low state anxiety (CAM-S score (mean ± SD): 2.2 ± 2.1). Males reported to spend more time on videogames, to perceive higher self-control and to be more influenced by reinforcement mechanisms; females described higher levels of trait anxiety. In the regression analysis, state anxiety was a predictor of videogame use and addiction (*p* = 0.01). Further research is needed to confirm these data and to maximize the developmentally positive effects of videogames and preventing the negative consequences.

## 1. Introduction

Over the last decade, the amount of time that children and adolescents spend online playing videogames has constantly increased [1]. Lockdowns, quarantine measures and physical distancing imposed by the COVID-19 pandemic led to a further spread of indoor activities, including online gaming [2].

The consequences of online videogame use depend on frequency and duration. Previous studies found positive effects of online gaming in children, including distraction from pre-operative anxiety [3,4], motivational treatment of obesity [5], autism [6,7,8], amblyopia [9,10], psychiatric disorders [11], non-pharmacological treatment of cancer-related symptoms [12], improvement of intrinsic motivation [13], visual attention [14,15], visuospatial ability and speed of navigation [16] and physical activity [17].

By contrast, other studies found a correlation between excessive and compulsive use of videogames and retreat, social problems, attention difficulties and criminal and aggressive behaviors [18,19,20]. Recent studies showed that even a brief exposure to violent videogames can increase aggressive cognition and aggressive behaviors, especially in males [21]. According to the United Nations Children’s Fund (UNICEF), millions of children are currently exposed to the growing risks associated with the virtual world [22].

Addictive behaviors towards playing online have been linked to a possible diagnosis of Internet Gaming Disorder (IGD) [1]. The Diagnostic and Statistical Manual of Mental Disorders, Fifth Edition (DSM-5), includes IGD in Section 3, as a new condition warranting more clinical research. According to the current definition, IGD is characterized by a “persistent and recurrent use of the Internet to engage in games, often with other players, leading to clinically significant impairment or distress”, as suggested by the presence of 5 (or more) diagnostic criteria within a 12-month period. The diagnostic criteria include preoccupation or obsession with gaming, withdrawal symptoms, tolerance, loss of control, loss of other interests, continued overuse despite negative consequences, deceptive behaviors in order to play, mood alterations and escape of negative feelings due to gaming, as well as work, relational and functional impairment [23]. More recently, the World Health Organization (WHO) included this gaming disorder in the International Statistical Classification of Diseases and Related Health Problems (ICD-11) [24].

Recent research documented the association between IGD and psychosocial variables in adolescents, including anxiety, depression, OCD, somatization and social difficulties [19,25,26]. Moreover, IGD was shown to be a significant contributor to the increase in sleep difficulties and impaired quality of life in adolescents during the COVID 19 outbreak, with anxiety being a strong mediator of the effects of IGD on sleep disturbances [2]. Parenting styles and family rules about videogaming were reported to be a significant factor in reducing the negative effects due to the use of videogames, in particular in terms of increased aggressivity and fighting behaviors [27].

A recent study [28] has provided data supporting the possibility of the existence of an optimal amount of time spent playing videogames that can at the same time maximize the possible benefits and be safe (i.e., not induce behavioral and emotional dyscontrol). However, this topic needs to be further studied, taking also into consideration variables related to psychopathological dysfunctions.

In this respect, anxiety can be defined as an alarm reaction to a stimulus perceived as dangerous, with the feeling that something bad is going to happen [23,29]. It represents a common reaction to stress. In 1961, Cattel and Scheier introduced the distinction between trait and state anxiety. Trait anxiety is a relatively stable individual personality characteristic, which refers to a general tendency to respond with anxiety and worry to perceived threats and dangers in the external environment. State anxiety reflects a transitory emotional state, characterized by feelings of tension and apprehension, with increased autonomic nervous system activity. When the subject experiences high state anxiety, he tries to limit these feelings through specific mechanisms, both psychological and behavioral [30].

According to the DSM 5, anxiety disorders include conditions that share features of excessive fear and anxiety and related behavioral disturbances, leading to an impairment in daily functioning [23]. A number of reports has shown that children were at risk of increased anxiety and anxiety-related behaviors during the COVID-19 pandemic, especially during the so-called “lockdown” [31]. To date, limited research specifically investigated the association between anxiety and videogames use in this age group [32]. Moreover, no epidemiological or quasi-epidemiological data have been published regarding the prevalence of videogames use and videogame addiction in Italy.

We conducted a cross-sectional study to assess the prevalence of videogames use and videogames addiction in a sample of Italian children during the COVID-19 pandemic. We also investigated the prevalence of trait and state anxiety symptoms, given their possible increase related to the COVID-19 pandemic, and their association to videogames use.

## 2. Materials and Methods

### 2.1. Recruitment

We recruited a sample of children in three primary schools in the province of Catania (Italy); all children were attending school in presence in the last three months (i.e., from September to November 2020). After discussing with the school heads and receiving their consent to the administration of the tests during school hours, we sent written information to all parents or legal guardians of children attending these primary schools, being available for face-to-face contacts but also to discuss the study and its characteristics by phone or messaging systems. After that, all parents or legal guardians signed an informed consent. In total, 162 children (78 males, 84 females), 8–10 years old (average; 9.4; SD: 0.7), were included in this study: 24.7% of them were an only child, while 75.3% had brothers or sisters.

### 2.2. Instruments

We administered an ad hoc questionnaire to explore the demographic variables (i.e., age, sex, having brothers or sisters), the access to electronic tools that could be used for playing and the games usually played.

Moreover, each child completed three questionnaires:

(1) the Videogame Addiction Scale for Children (VASC) [1]: specifically designed for children, this is a self-administered 21-item questionnaire investigating a possible addiction to videogames. All items are scored on a 5-point scale (from never to very often). The test provides a total score (range 21–105), and 4 sub-scores, related to four psychological dimensions (self-control, reinforcement, life-style and time-use problems and game involvement). A global score above 90 indicates the possibility of videogame addiction. The scale proved a Cronbach’s alpha of 0.898 in our study sample;
(2) the Test of Anxiety and Depression (TAD) [33]: this is a self-report questionnaire investigating anxiety and depression. In the present study, we administered only the 11 items related to trait anxiety, in order to reduce the number of questions to be answered and improve children’s collaboration and attention. The score obtained (range: 55–150) was classified as follows: anxiety symptoms below average (99 or lower), average non pathological (100–114), mildly pathological (115–129), moderately pathological (130–144) and highly pathological (145 or higher). The Cronbach’s alpha in our study sample was 0.822;
(3) the Children’s Anxiety Meter—State (CAM-S) [34]: this is a validated visual analogue scale, drawn to resemble a thermometer with ten levels. To measure state anxiety (CAM-S), children are asked to mark how they feel “right now”. The Cronbach’s alpha was 0.867 in our study sample.

### 2.3. Data Analysis

Statistical analysis was performed using IBM SPSS Statistics 26 for Windows.

### 2.4. Ethical Approval

Ethical approval for the study was granted by the Ethic committee of the Department of Education Science, University of Catania.

## 3. Results

Overall, 96.3% of the 162 children recruited reported access to one or more devices used to play videogames (console (65.4%), smartphone (56.2%), tablet (48.1%) or personal computer (36.4%)). Their favorite games were Fortnite (21.6%), FIFA (10.5%), Minecraft (7.4%), SuperMario (6.2%) and Just Dance (4.3%).

We found an average score at the VASC of 46.7 (SD: 15.4), indicating a low risk of videogame addiction; the average score at the TAD was 135 (SD 16.8), indicating a moderate level of pathological trait anxiety; as for the CAM-S, a low level of state anxiety was reported (average 2.2, SD 2.1).

There were significant differences comparing males and females (Table 1). Despite not being in general at risk for videogame addiction (VASC global score below 90), males used videogames more than females (t = 4.06; *p* < 0.001), declared higher self-control (t = 3.63, *p* < 0.001) and had a higher level of reinforcement mechanisms (t = 4.36; *p* < 0.001), but also of trait anxiety (TAD score; t = −5.18, *p* < 0.001).

The correlation analysis showed in the whole sample that state anxiety was positively related to videogames use (*r* = 0.19; *p* < 0.05), reinforcement mechanisms (*r* = 0.21; *p* < 0.01) and involvement in videogames use (*r* = 0.17; *p* < 0.05), while we found no significant correlations between trait anxiety levels and the VASC score or sub-scores. Analyzing males, state anxiety had a positive correlation with videogame involvement (*r* = 0.26; *p* < 0.05), while trait anxiety correlated with more frequent use of videogames (*r* = 0.34; *p* < 0.01), problems with life-style and time-use (*r* = 0.27; *p* < 0.05) and self-control (0.26; *p* < 0.05). As for females, state anxiety was positively related to videogames use (*r* = 0.22; *p* < 0.05) and with reinforcement mechanisms (*r* = 0.35; *p* < 0.01), while trait anxiety correlated with videogames use (*r* = 0.31; *p* < 0.01), reinforcement mechanisms (0.27; *p* < 0.05) and involvement in videogames (*r* = 0.28; *p* < 0.05).

We also conducted two linear regression analyses with the enter method between a dependent variable (VASC) and two independent variables (TAD and CAM) on the whole sample. They showed that state anxiety (standardized beta 0.152; t = 1.94; *p* = 0.01) was a possible predictor of videogame use and addiction, while trait anxiety was only “nearly significant” (standardized beta 0.192, t = 2.47; *p* = 0.054).

## 4. Discussion

Children in our sample showed on average a low risk of videogame addiction, a moderate level of trait anxiety and a low state anxiety. We found significant gender-related differences: males tend to spend more time on videogames, were perceived to have a higher self-control and to be more influenced by reinforcement mechanisms; females describe higher levels of trait anxiety. State anxiety emerged from the regression analysis as a risk factor for problematic videogame use in these children.

The mean VASC scores in our study were similar to those reported by Ylmaz et al. (2017), in a sample of 780 school children before the COVID-19 pandemic [1]. Moreover, in line with previous research, boys reported higher scores compared to girls [1,25].

To date, there is still an ongoing debate about the proper conceptualization of IGD [35,36]. Videogame playing has become one of children’s favorite leisure activities. Previous research emphasized that the classification of videogaming behavior as an addiction or mental disorder can be problematic since excessive use of videogames concerns mainly adolescents and young adults, with a high rate of spontaneous remission [25]. The positive or negative consequences of videogames for child development have been widely debated in the scientific literature [5,36]. Videogames can have a number of positive effects, being well-accepted methods to stimulate motor, attentive and perceptual skills, but also motivation towards a goal and social skills [4,37].

The existing literature has focused on five aspects to be considered to assess the effect of videogames on children: the amount of time spent playing (especially if without pauses and with a complete immersion in the videogame), the content of the videogame, the game context, the game structure and the game mechanics [5,35]. Videogames can lead to unhealthy behaviors, including a sedentary lifestyle (with the consequent risk of developing over-weight), a reduction of time dedicated to academic learning (or a lack of concentration in performing school duties) and the substitution of all other forms of social relations with the videogame (favoring a state of isolation and a tendency towards introversion) [32,38,39,40].

Moreover, a large body of research clearly documented the association between IGD and anxiety symptoms in adolescents. In this regard, previous studies showed how videogames may be used as a treatment tool for internalized anxiety [41,42,43] while others highlighted that anxiety could be the reason for excessive videogames use, or vice versa [29,32]. According to the comorbidity hypothesis, IGD shares common psychological and neurobiological features (including craving) with other addictive behaviors and psychiatric problems. The experience of persistent high levels of anxiety could lead to problematic videogaming in order to avoid negative affective states, and this behavior could be maintained through reinforcement mechanisms [25,44].

Regarding gender differences, previous studies found that an increased amount of time spent playing with videogames was associated with higher anxiety symptoms in female adolescents. By contrast, it was associated with decreased anxiety levels in males [42,43]. Social support may represent a probable explanation, given the important differences in terms of the “social connection” that videogames seem to provide to boys compared to girls. Of note, male adolescents, while playing, seem to interact and create new friends much more frequently than females [45]. In our sample, male children perceived to have a higher self-control and to be more influenced by reinforcement mechanisms.

## 5. Conclusions

Our data are relevant to help children and caregivers to be aware of the risk of developing addiction to videogames [41]. Of note, males tend to perceive themselves as more “in control” of videogames use. Caregivers should define rules for videogaming, in terms of duration and of access to the devices [46]. Finally, given the role of state anxiety as a risk factor for problematic videogame use, it is important to monitor anxiety symptoms and their intensity in children as a preventive strategy.

Our study has some limitations. First, its cross-sectional design does not allow to understand the evolution of the relationship between anxiety and videogaming. Second, children were recruited during the so-called “second wave” of the SARS-CoV-2 diffusion in Italy, a fact that could have increased the reported levels of anxiety. Third, we used only self-administered questionnaires, a fact that could limit the interpretation of our results. Specifically, we assessed IGD and anxiety symptoms. Only a detailed clinical assessment can identify IGD and anxiety disorders. Fourth, the sample size was relatively small; therefore, our data should be replicated on larger samples. In fact, the number of children recruited is limited and could therefore be insufficient to represent the population of subjects of the same age range.

Notwithstanding these limitations, this study provides information about an emergent important issue in children in the context of a highly stressful and unique situation, such as the COVID-19 pandemic. Recently, a possible use of active video games for improving mental health and physical fitness during isolation periods was reported [47]; however, more studies are needed to define the most adequate interventions to be activated by caregivers to prevent the negative consequences and maximize the developmentally positive effects of videogames [14,48].

## Figures and Tables

**Table 1:** Differences comparing males and females in the Videogame Addiction Scale for Children (VASC), the Children’s Anxiety Meter—State (CAM-S) and the Test of Anxiety and Depression (TAD).

	Male		Female			
	M	SD	M	SD	T	*p*
Self-control	7.95	2.43	6.59	2.31	3.637	<0.001
Reinforcement mechanisms	11.90	4.63	9.11	3.46	4.363	<0.001
Problems	9.68	3.72	8.30	3.51	2.435	0.016
Involvement in videogames	2.17	1.36	1.89	1.13	1.396	0.165
VASC	51.63	16.58	42.20	12.79	4.068	<0.001
CAM-S	2.24	2.40	2.23	1.71	0.053	0.957
TAD	14.77	3.88	18.81	5.76	−5.183	<0.001

## Data Availability

Raw data are available from the corresponding author upon reasonable request.
